# Risk Factors for Progression from Severe Maternal Morbidity to Death: A National Cohort Study

**DOI:** 10.1371/journal.pone.0029077

**Published:** 2011-12-28

**Authors:** Gilles Kayem, Jennifer Kurinczuk, Gwyneth Lewis, Shona Golightly, Peter Brocklehurst, Marian Knight

**Affiliations:** 1 National Perinatal Epidemiology Unit, University of Oxford, Oxford, United Kingdom; 2 Policy Research Unit in Maternal Health and Care, University of Oxford, Oxford, United Kingdom; 3 Department of Health, London, United Kingdom; 4 Centre for Maternal and Child Enquiries, London, United Kingdom; The University of Adelaide, Australia

## Abstract

**Background:**

Women continue to die unnecessarily during or after pregnancy in the developed world. The aim of this analysis was to compare women with severe maternal morbidities who survived with those who died, to quantify the risk associated with identified factors to inform policy and practice to improve survival.

**Methods and Findings:**

We conducted a national cohort analysis using data from two sources obtained between 2003 and 2009: the Centre for Maternal and Child Enquiries maternal deaths database and the United Kingdom Obstetric Surveillance System database. Included women had eclampsia, antenatal pulmonary embolism, amniotic fluid embolism, acute fatty liver of pregnancy or antenatal stroke. These conditions were chosen as major causes of maternal mortality and morbidity about which data were available through both sources, and include 42% of direct maternal deaths over the study period. Rates, risk ratios, crude and adjusted odd ratios were used to investigate risks factors for maternal death. Multiple imputation and sensitivity analysis were used to handle missing data.

We identified 476 women who survived and 100 women who died. Maternal death was associated with older age (35+ years aOR 2.36, 95%CI 1.22–4.56), black ethnicity (aOR 2.38, 95%CI 1.15–4.92), and unemployed, routine or manual occupation (aOR 2.19, 95%CI 1.03–4.68). An association was also observed with obesity (BMI≥30 kg/m^2^ aOR 2.73, 95%CI 1.15–6.46).

**Conclusions:**

Ongoing high quality national surveillance programmes have an important place in addressing challenges in maternal health and care. There is a place for action to reverse the rising trends in maternal age at childbirth, and to reduce the burden of obesity in pregnancy, as well as ongoing recognition of the impact of older maternal age on the risks of pregnancy. Development and evaluation of services to mitigate the risk of dying associated with black ethnicity and lower socioeconomic status is also essential.

## Introduction

Globally, reducing maternal mortality has been recognised as an important challenge facing all governments and international agencies [1]. More than 350,000 women are estimated to die annually during or shortly after pregnancy worldwide, and although this has decreased by more than a third from the estimated figure in 1990 [2], the rate of decline is less than half of that required to reach the target of the United Nations Millennium Development Goal 5: to reduce the maternal mortality ratio (MMR) by three quarters between 1990 and 2015 [1], [3]. Although the greatest challenges in tackling maternal mortality face the developing world, women continue to die unnecessarily during or after pregnancy in the developed world [4], [5]. Maternal mortality rates, in general, are not declining in the developed world, and indeed, in some countries, such as the US, have doubled over the last 20 years [2].

Nevertheless, because maternal deaths in the developed world are still uncommon, identifying factors that can be addressed to prevent death may be difficult precisely because cases are rare. Comprehensive and lengthy surveillance is needed to generate sufficient information to guide changes in policy or practice. It is increasingly being recognised that the additional study of severe maternal morbidity can complement enquiries into maternal deaths and is therefore of increasing importance to service providers and policymakers in the area of maternal health [6]. Cases are more frequent, studies can be conducted more quickly and conclusions are statistically more robust. Importantly, morbidity and mortality cases can be compared in order to investigate factors associated with progression to death and hence lead to actions aimed at improving survival. A considerable body of research conducted in both developed and developing country settings has focused on the analysis of severe maternal morbidity cases with the aim of understanding and addressing health system failures in obstetric care [7]. However, very few countries have comprehensive surveillance systems to identify and investigate both maternal mortality and severe morbidity cases. The UK has had a detailed confidential enquiry into maternal deaths for approaching 60 years [4]; the recent introduction of the UK Obstetric Surveillance System (UKOSS) to study specific causes of severe maternal morbidity uniquely allows for comparison of morbidity and mortality cases on a national population basis [8].

The aim of this analysis was to compare the characteristics of women with a range of specific severe maternal morbidities who survived with those who died to quantify the risks associated with identified factors in order to inform policy and practice to improve survival.

## Methods

### Ethics Statement

The London Multi-centre Research Ethics Committee approved the UKOSS general methodology (04/MRE02/45) and the studies of individual severe morbidities (04/MRE02/46, 04/MRE02/71, 04/MRE02/72, 04/MRE02/73, 07/H0718/54). Surveillance of maternal death through CMACE is a form of national audit and does not require Research Ethics Committee Approval. Collection of data by CMACE was approved by the National Information Governance Board.

### Data collection

Data concerning women who died and women who survived from five specific maternal conditions were analysed: eclampsia, antenatal pulmonary embolism, amniotic fluid embolism, acute fatty liver of pregnancy, and antenatal cerebral stroke. These conditions were chosen for the pragmatic reason that they represent major causes of maternal mortality and morbidity about which data were available through both UKOSS and the confidential enquiry into maternal deaths carried out by the Centre for Maternal and Child Enquiries (CMACE). Data for the analysis were obtained from two separate sources: information about women who died was obtained from the CMACE confidential enquiries into maternal death database and information about women with severe morbidity who survived was obtained from the UKOSS database.

### UKOSS data collection

Cases of severe maternal morbidity for these purposes were defined as women with eclampsia, antenatal pulmonary embolism (PE), amniotic fluid embolism (AFE), acute fatty liver of pregnancy (AFLP) and antenatal stroke (for definitions see Methods S1), and were identified through the monthly UKOSS mailing between 2005 and 2009. Details of the specific time periods of surveillance for each condition are shown in Table 1. UKOSS is an active, negative surveillance system with a rolling programme of studies, covering all hospitals with consultant-led maternity units in the UK, and thus effectively covers the entire cohort of women delivering [8]. We sent UKOSS case notification cards every month to nominated reporting clinicians in each hospital in the UK with a consultant-led maternity unit, with a tick box list to indicate whether they had seen any women, including women who died, with the conditions under study. We also asked them to return cards indicating a ‘nil report’, so that we could monitor cards return rates and confirm the denominator maternity population to calculate the incidence. In the UK, women may also deliver in midwifery-led units or at home (in total approximately 3–6% of births); however, any women in one of these settings with a severe maternal morbidity will always be transferred to a consultant-led unit. When a clinician returned a card indicating an eligible case, the reporting clinician was sent a data collection form requesting further details of prognostic factors, management and outcomes. Up to five reminders were sent if completed forms were not returned. Data collection sheets for cases were checked to confirm that they met the case definitions (Methods S1).

**Table 1:** Number of women who died and number who survived from specific causes of severe maternal morbidity.

	Data source	Start of data collection	End of data collection	Duration of data collection (months)	Number of Cases (%)	Estimated number of maternities†
**Mortality** * **:**						
All deaths	CMACE	01/01/2003	31/12/2005	36	60 (60)	2,114,004
All deaths	CMACE	01/01/2006	31/12/2008	36	40 (40)	2,291,493
**TOTAL DEATHS**					**100 (100)**	
**Morbidity** ** **:**						
Amniotic fluid embolism	UKOSS	01/03/2005	28/02/2009	48	48 (10)	3,032,560
Acute fatty liver of pregnancy	UKOSS	01/02/2005	31/08/2006	19	56 (12)	1,147,848
Antenatal pulmonary embolism	UKOSS	01/02/2005	31/08/2006	19	138 (29)	1,147,848
Eclampsia	UKOSS	01/02/2005	28/02/2006	13	214 (45)	779,442
Antenatal stroke	UKOSS	01/07/2007	31/12/2008	18	20 (4)	1,168,233
**TOTAL SURVIVORS**					**476 (100)**	

*Maternal deaths from eclampsia (n = 14), antenatal pulmonary embolism (n = 28), amniotic fluid embolism (n = 30), acute fatty liver of pregnancy (n = 3) and antenatal stroke (n = 25).

† Data from Office for National Statistics, Key Population and Vital Statistics. [34].

**Number of women after exclusion of fatal cases for the five morbidities.

Cases for UKOSS studies were additionally ascertained by contacting clinicians in intensive care units, specialist liver units and radiology departments, as appropriate. Very few additional cases (n = 3) were identified through these additional sources. Maternal deaths were also ascertained through UKOSS, and routine cross-checking with CMACE data showed a high level of case ascertainment. However, for the purposes of this analysis, all maternal death cases were excluded from UKOSS data to ensure these data were limited solely to cases of morbidity where the woman survived.

### CMACE data collection

The methodology of the confidential enquiries into maternal deaths has been described in detail previously [9]. In summary, cases of maternal death are reported to CMACE through several different sources and, in addition, ascertainment of cases is undertaken through linkage of routine birth and death vital statistics records. Cases of maternal death from eclampsia, antenatal pulmonary embolism, amniotic fluid embolism, acute fatty liver of pregnancy and antenatal stroke occurring between 2003 and 2008 were identified by interrogating the CMACE database and contacting the clinicians on the confidential enquiry panel responsible for assessing the cause of death.

UKOSS data were collected during different durations and time periods from CMACE. However all the UKOSS data were collected during the 2003–2008 CMACE data collection period. As maternal deaths are rare, we took the approach of using the CMACE data available over the full time period in order to maximize the statistical power of these analyses.

### Statistical analyses

The incidence of maternal mortality and severe maternal morbidity and the ratio of survivors to deaths were calculated for each condition with 95% confidence intervals (CI). The denominator used was the number of maternities (women delivering one or more live or stillborn infants) during each study period, estimated from the most appropriate UK birth data available (Table 1) [10].

In order to investigate trends in continuous variables, unadjusted relative risks with 95% CIs were calculated across groups and Spearman correlation was used to examine the associations between age and BMI and the risk of death. We further investigated the potential factors underlying mortality differences in severe maternal morbidities by using a logistic regression analysis. Factors were included where there was a pre-existing hypothesis or evidence to suggest that they may be associated with maternal mortality. In order to adjust for any effect relating to the individual morbidities, we included an adjustment factor for each condition in the analysis. We developed a full regression model by including both potential explanatory and confounding factors. We tested continuous variables for departure from linearity by the addition of quadratic terms to the model and subsequent likelihood ratio testing. We calculated adjusted odds ratios with 95% CI.

The factors included in the model were maternal age, parity, body mass index (BMI), smoking during pregnancy, ethnicity, and socioeconomic classification based on occupation. Occupation was classified according to the Office for National Statistics socio-economic classification [11], on the basis of the woman's occupation, unless she was not in paid employment, in which case the occupation of her partner was used. Ethnicity was categorised according to the UK census classification [12].

Data were missing for ethnicity and BMI for between 12% and 23% of cases. We investigated two different methods of analysis to account for this. In a first analysis we included all participants, with creation of a categorical indicator variable for missing responses (missing indicator). The second analysis included all participants with missing responses by using multiple imputation. Missing data for BMI, type of employment, age, parity, smoking and ethnicity were imputed using chained equations [13], [14]. The multiple imputation prediction model included all variables in the conceptual framework. In addition, indicator variables for the following characteristics were included in the prediction model: cause of death or morbidity and route of recruitment into the analysis (CMACE or UKOSS). Twenty imputed data sets were created and analyzed together. Standard logistic regression models were fitted using Stata 10. The imputed data sets were analyzed in Stata 10 using the ice suite of commands [13].

Maternal pre-existing physical and psychiatric conditions were reported only for the 2006–08 CMACE data. There were additionally fewer missing data for these women (7% missing for BMI, 20% missing for occupation). We therefore repeated the multivariate analyses with both missing indicator and multiple imputation models in a subset analysis, using only data on the population of women who died from 2006 to 2008 (n = 40) and the women who had a severe maternal morbidity and survived (n = 476) to investigate the role of pre-existing maternal conditions in death following severe morbidity.

In addition, we assessed the robustness of the analysis, given the limitation of the missing data, by undertaking a series of sensitivity analyses. In each case we assumed specific extreme scenarios and apportioned missing values accordingly.

We assessed the additive effect of the presence of multiple risk factors on the risk of death in a model including all factors found to be significantly associated. The final model included maternal age over 30, BMI equal or over 30 kg/m^2^, black Caribbean or African ethnicity and unemployed, routine or manual occupation.

We used Stata version 10 software for all analyses (StataCorp, College Station, TX).

## Results

One hundred maternal deaths from one of the five specific causes under investigation were identified from the CMACE database to have occurred between 2003 and 2008. A total of 476 women with severe morbidity who survived were identified through UKOSS (Table 1).

The estimated mortality and morbidity rates are shown in Table 2. Among the five conditions, the ratio of survivors to deaths was lowest for AFE, stroke and PE: for every two women with an AFE who die, five have an AFE and survive; for every woman dying of a stroke, three survive; and for every woman dying of a PE 19 survive. The ratio of survivors to deaths was highest for AFLP and eclampsia: for every woman dying of AFLP, 70 survived and for every woman dying of eclampsia 86 women survived.

**Table 2:** Incidence of death and survival, and the ratio of survivors to deaths for specific causes of severe maternal morbidity.

	Incidence of maternal death per 1 000 000 maternities (95%CI)	Incidence of survival from severe morbidity per 1 000 000maternities (95%CI)	Ratio of survivors to deaths (95%CI)
Amniotic fluid embolism	6.8 (4.6–9.7)	15.8 (11.7–21.0)	2.3∶1 (1.2∶1 to 4.6∶1)
Acute fatty liver of pregnancy	0.7 (0.1–2.0)	48.8 (36.9–63.4)	70∶1 (18∶1 to 634∶1)
Antenatal pulmonary embolism	6.4 (1.4–9.2)	120 (101–142)	19∶1 (11∶1 to 101∶1)
Eclampsia	3.2 (1.7–5.3)	275 (239–314)	86∶1 (45∶1 to 185∶1)
Antenatal cerebral stroke	5.7 (3.7–8.4)	17.1 (10.5–26.4)	3.0∶1 (1.3∶1 to 7.1∶1)

Numerator data obtained from UKOSS and CMACE databases and denominator data from the Office for National Statistics, Key Population and Vital Statistics 2007. Office for National Statistics, Newport.

**Figure 1:**
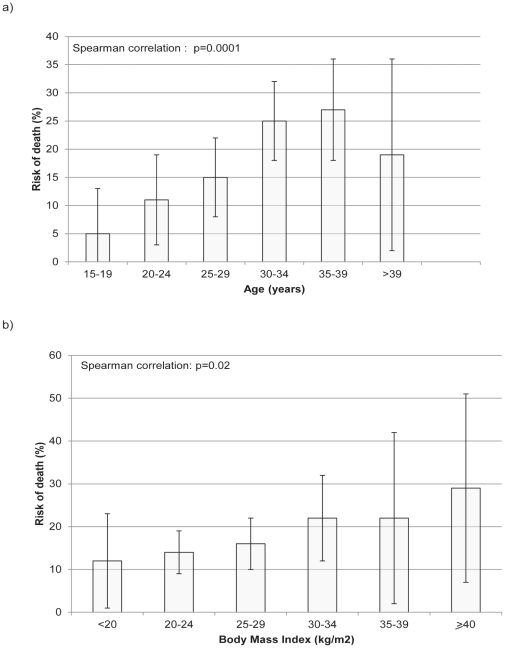
Risk of death according to a) age and b) body mass index (bars show risks with 95% confidence intervals).

Increasing maternal age and body mass index were significantly associated with the risk of death (Figure 1). Results from the multivariable models are presented in Table 3. The final model using a missing indicator analysis produced very similar effect estimates to those from the multiple imputation analysis. Women who were over 30, black Caribbean or African, unemployed or with routine or manual occupation had higher odds of progressing to death (Table 3). Women who had a BMI equal to or greater than 30 kg/m^2^ were more likely to die from thesesevere maternal morbidities (aOR 1.71, 95%CI 0.91–3.19) although the increased odds were not statistically significant in the model including all maternal deaths.

**Table 3:** Factors associated with the odds of death from specific causes of maternal morbidity.

	Maternal deaths n = 100, n(%)	Survivors n = 476, n(%)	Crude OR* (95%CI)	Adjusted OR* Missing indicator model (95%CI)	Adjusted OR* multiple imputation model (95%CI)
Age (years)					
<30	32 (32)	269 (57)	1	1	1
30–34	40 (40)	120 (25)	2.80 (1.68–4.68)	2.89 (1.56–5.36)	2.58 (1.36–4.90)
≥35	27 (27)	85 (18)	2.67 (1.51–4.71)	2.04 (1.01–4.11)	2.36 (1.22–4.56)
Missing	1 (1)	1 (0)			
Parity					
Nulliparous	46 (46)	286 (60)	1	1	1
Multiparous	53 (53)	189 (40)	1.74 (1.13–2.70)	0.75 (0.44–1.28)	0.76 (0.45–.1.29)
Missing	1 (1)	1 (0)			
Ethinicity					
White	65 (64)	352 (74)	1	1	1
Black Caribbean and African	19 (20)	45 (9)	2.44 (1.34–4.44)	2.40 (1.14–5.06)	2.38 (1.15–4.92)
Other minority ethnic groups	15 (15)	72 (15)	1.20 (0.64–2.23)	1.16 (0.54–2.51)	1.31 (0.65–2.76)
Missing	1 (1)	7 (1)			
BMI (kg/m^2^)					
<30	53 (53)	327 (67)	1	1	1
≥30	24 (24)	84 (18)	1.83 (1.06–3.14)	1.57 (0.83–2.97)	1.71 (0.91–3.19)
Missing	23 (23)	65 (14)			
Occupational classification					
Managerial	20 (25)	113 (24)	1	1	1
Intermediate occupation	19 (23)	101 (21)	1.06 (0.54–2.10)	1.41 (0.65–3.07)	1.33 (0.59–2.95)
Manual or unemployed	42 (52)	207 (43)	1.14 (0.64–2.05)	2.33 (1.13–4.80)	2.19 (1.03–4.68)
Missing	19 (19)	55 (12)			
Smoking during pregnancy					
Yes	13 (14)	89 (19)	0.67 (0.35–1.26)	0.62 (0.29–1.32)	0.58 (0.27–1.26)
No	82 (86)	376 (79)	1	1	1
Missing	5 (5)	9 (2)			

*Odds of death.

In the analysis restricted to the 2006–8 maternal death data, 107 (22%) of the women who survived had a pre-existing medical condition compared with nine (22%) of the women who died (Table 4). Fifteen percent of the women who died had reported pre-existing depressive illness versus three percent of survivors (p<0.001). Three women who died had learning disabilities compared with none of the women who survived (p<0.001). To explore the role of pre-existing medical conditions further, we undertook a second multivariable analysis including maternal age, smoking, parity, BMI, ethnicity, occupational status and the presence of pre-existing disease (Table 5). Odds ratio estimates of the risk of death were not significantly different using missing indicator or multiple imputation models. In the multiple imputation model, maternal death was associated with a BMI≥30 kg/m^2^ [aOR 2.73, 95%CI 1.15–6.46], maternal age of 30–34 [aOR 4.29, 95%CI 1.63–11.2] or ≥35 [aOR 5.26, 95%CI 1.86–14.5] and black Caribbean or African ethnicity [aOR 3.01, 95%CI 1.08–8.42]. Women from unemployed or routine or manual occupational groups appeared to be more likely to progress to death [aOR 3.27, 95%CI 1.03–10.4]. The presence of a pre-existing medical or mental health condition was not associated with a higher odds of death [aOR 0.88, 95%CI 0.39–1.99].

**Table 4:** Pre-existing medical conditions in women who died (2006–8 period) and those who survived.

	Women who died	Survivors
	n = 40 n (%)	n = 476 n (%)
Previous history		
Cancer	0	3 (1)
Previous Thrombotic event	0	3 (1)
Current disease		
Asthma	1 (2)	39 (8)
Auto immune disease	1 (2)	3 (1)
Congenital or aquired Cardiac disease	1 (2)	7 (1)
Diabetes, endocrine disorders	2 (5)	12 (2)
Epilepsy	1 (2)	3 (1)
Essential hypertension	3 (7)	8 (2)
Haematological disorders	0	5 (1)
Renal or urological disease	0	5 (1)
Others	0	19 (4)
Overall	9 (22)	107 (22)

Note that some women had more than one condition.

**Table 5:** Factors associated with the odds of death from specific causes of maternal morbidity (Analysis limited to 2006–8 data only).

	Maternal deaths n = 40, n(%)	Survivors n = 476, n(%)	Crude OR* (95%CI)	Adjusted OR* multiple imputation model (95%CI)
Age (years)				
<30	10 (25)	269 (57)	1	1
30–34	11 (27)	120 (25)	2.85 (1.17–6.90)	4.29 (1.63–11.2)
≥35	19 (48)	85 (18)	4.96 (2.23–11.0)	5.26 (1.86–14.5)
Missing	0	1 (0)		
Parity				
Nulliparous	19 (48)	286 (60)	1	1
Multiparous	20 (50)	189 (40)	1.59 (0.83–3.06)	0.62 (0.28–1.35)
Missing	1 (2)	1 (0)		
Ethinicity				
White	26 (65)	352 (74)	1	1
Black Caribbean and African	8 (20)	45 (9)	2.41 (1.03–5.64)	3.01 (1.08–1.42)
Other minority ethnic groups	6 (15)	72 (15)	1.13 (0.45–2.84)	1.49 (0.50–4.45)
Missing	0	7 (1)		
BMI (kg/m^2^)				
<30	24 (60)	327 (67)	1	1
≥30	13 (33)	84 (18)	2.11 (1.03–4.32)	2.73 (1.15–6.46)
Missing	3 (7)	65 (14)		
Occupational classification				
Managerial	7 (17)	113 (24)	1	1
Intermediate occupation	8 (20)	101 (21)	1.28 (0.45–3.65)	1.80 (0.59–5.55)
Manual or unemployed	17 (43)	207 (43)	1.33 (0.54–3.29)	3.27 (1.03–10.4)
Missing	8 (20)	55 (12)		
Smoking during pregnancy				
Yes	7 (20)	89 (19)	1.06 (0.45–2.50)	1.06 (0.36–3.13)
No	28 (70)	376 (79)	1	1
Missing	5 (20)	9 (2)		
Pre-existing condition				
Yes	9 (22)	89 (19)	0.83 (0.39–1.80)	0.88 (0.39–1.99)
No	31 (78)	376 (79)	1	1
Missing	0	9 (2)		

*Odds of death.

To explore the effects of potential biases related to the missing values in the model including all the women who died and survived, we tested the sensitivity of our multivariable model to a series of assumptions about the missing values (Table 6). Women who had a manual occupation or were unemployed, and those who were black Caribbean or African had higher odds of death in all the different analyses used to account for the missing data. The association of women's BMI with maternal death, although resulting in similar odds ratio estimates whatever the hypothesis used to account for the missing values, was inconstantly statistically significant.

**Table 6:** Adjusted odds ratios by missing data values: result of sensitivity analyses.

*Maternal characteristics*	*Missing value scenario assumptions*	*aOR (95%CI)* *
BMI (kg/m^2^)	All missing BMI values assumed to be <30 kg/m^2^	
<30		1
≥30		1.30 (0.70–2.40)
BMI (kg/m^2^)	All missing BMI values assumed to be ≥30 kg/m^2^	
<30		1
≥30		1.99 (1.17–3.36)
BMI (kg/m^2^)	Restricted to the period 2006–08 for CMACE data. All missing BMI values assumed to be <30 kg/m^2^	
<30		1
≥30		2.60 (1.11–6.12)
BMI (kg/m^2^)	Restricted to the period 2006–08 for CMACE data. All missing BMI values assumed to be ≥30 kg/m^2^	
<30		1
≥30		1.65 (0.75–3.63)
Ethnicity	All missing ethnicities assumed to be white	
White		1
Black African or Caribbean		2.25 (1.07–4.71)
Indian, Bangladesh, Pakistan, Other minorities		1.08 (0.50–2.30)
Ethnicity	All missing ethnicities assumed to be Black Caribbean or African	
White		1
Black African or Caribbean		2.55 (1.27–5.11)
Indian, Bangladesh, Pakistan, Other minorities		1.16 (0.54–2.49)
Employment	All missing occupational codes assumed to be managerial	
Managerial		1
Non managerial		1.17 (0.58–2.36)
Manual or unemployed		1.85 (1.01–3.40)
Employment	All missing occupational codes assumed to be manual or unemployed	
Managerial		1
Non managerial		1.41 (0.65–3.06)
Manual or unemployed		2.14 (1.07–4.27)

*adjusted for age, parity, ethnicity, BMI, employment and smoking.

Analysis of the combined effects of the risk factors present showed that the odds of death associated with these severe maternal morbidities increased progressively in the presence of more than one of the risk factors identified (table 7), although of note was the high degree of uncertainty around the estimated odds associated with the presence of all four risk factors.

**Table 7:** Odds ratio associated with death in cases of severe maternal morbidity according to the number of risk factors present.

Number of risk factors present	OR [95%CI]
0	1
1	1.35 (0.67–2.75)
2	2.77 (1.33–5.76)
3	4.40 (1.76–11.0)
4	8.45 (0.49–149)

Risk factors included: age ≥30; unemployment, routine or manual occupation; black Caribbean or African ethnicity; and a BMI equal or over 30 kg/m^2^.

## Discussion

This is the first study to compare on a national basis the characteristics of women who die in pregnancy with those who survive after experiencing one of a series of severe maternal morbidities. This analysis is uniquely possible because of the systems that exist in the UK to collect information about these women [4], [8]. Although previous maternal death reports have suggested that women with some of these characteristics are at higher risk of dying [9], these reports have not been able to quantify or assess independent risk factors associated with death. In the context of continuing concerns about maternal mortality [2], [3], this demonstrates the benefits of maintaining surveillance of both mortality and severe morbidity in high resource as well as low resource settings. Our analysis shows that in the UK, women who are older, obese, from manual or unemployed occupational groups and of black Caribbean or African ethnicity are more likely to die from these specific morbidities than women who are young, not obese, from other occupational groups and from white or other ethnic minority groups; the presence of two or more of these factors more than doubles the odds of death. Our findings highlight key areas for public health action and service provision.

Previous analyses have shown, independently, that women from minority ethnic groups are more likely to suffer from a severe morbidity in pregnancy [15] and more likely to die in pregnancy, particularly if they are recent immigrants [4], [16], [17]; this risk diminishes for the following generation [18]. The causes for this increase are not known, but language barriers, lack of familiarity with the health care system, cultural norms, and psychological problems, due for instance to events which occurred in their country of origin, including conflict and sexual assault, may all lead to difficulties in accessing antenatal care [4]. Moreover, poor communication between women and caregivers may result in inadequate care because of undiagnosed early symptoms or poor treatment compliance. In the Netherlands, the most important factors contributing to substandard care of immigrant women who die in pregnancy have been shown to be a delay in recognising symptoms and in referral by the general practitioner [19]. These findings highlight once more the importance of providing culturally sensitive antenatal services with the ready availability of interpreters, facilitating early self-referral and diagnosis for immigrant and ethnic minority women with pregnancy complications, particularly those from Black Caribbean or African groups.

There is a growing trend in developed countries for childbearing to occur at a later time in women's lives [20], [21]. The influence of increased age on maternal mortality has been clearly observed [4], [16], [22]. The most frequently cited explanation for the observed increase in mortality with age is that older women are more likely to suffer from co-existing disease which leaves them with less physiological reserve to cope with the additional insult of morbidity during pregnancy. However, we found no such association in our analysis; a fifth of both survivors and those who died had at least one significant past or pre-existing medical condition when they became pregnant. Thus whilst a pre-existing medical condition may increase the risk of developing severe maternal morbidity, this was not associated with an increased likelihood of dying. Furthermore, the increased risk of progressing from severe morbidity to death associated with age persisted after adjustment for known pre-existing medical conditions. It is possible that some of these women had undiagnosed pre-existing diseases contributing to death, since late or no antenatal care has been reported as a factor associated with maternal death [4]. However, it seems likely that older maternal age confers a health disadvantage, reflecting a lack of physiological robustness to respond to pregnancy pathology which is not fully appreciated by women or their clinicians. There is a clear need for public health action to reverse the rising trend in maternal age at childbirth by highlighting to clinicians, women and their partners the implications of decisions to delay child-bearing. In the meantime, clinical services need to appreciate that whilst older maternal age is now common, older mothers remain at higher risk.

Obese women were also more likely to die following severe maternal morbidity in our analysis. The analysis was limited by missing data but the association of obesity with death was significant when the analysis was performed including only the group of women who died between 2006 and 2008, when the data were more complete, even after adjustment for the presence of pre-existing medical conditions. Obesity is an increasingly important public health problem throughout the developed world. Obese pregnant women generally require care from a wide range of health professionals, have more complex pregnancies and require more interventions [23]. They also need specific high weight capacity equipment which is not necessarily widely available [23]. Critical care, which may be urgently needed in cases of severe maternal morbidity, can be particularly challenging in obese women. Anatomic changes associated with obesity can lead to specific difficulties related to emergency intubation and mechanical ventilation or catheterization procedures [24]. Obesity has been clearly shown to impact on mortality and morbidity associated with other complications in pregnancy, for example in the context of the recent AH1N1 influenza pandemic [25], [26]. Together these data serve to reinforce the need to address the problem of obesity in pregnancy; a wider perception of the additional pregnancy risks associated with obesity may provide an additional incentive for obese women who are planning pregnancy to lose weight. Currently evidence on the risks or benefits of weight loss or maintenance during pregnancy, and the optimal methods to encourage weight loss prior to, during or following pregnancy is lacking, and there is an urgent need for further research in this area. In the interim, appropriate hospital facilities should be available for obese pregnant women, and their care should be carefully planned, involving the multidisciplinary team, in order to evaluate and prevent additional complications.

Few studies have compared socioeconomic differences in mortality according to the cause of death [27], and none have studied risk factors for maternal death amongst women with severe maternal morbidity. Members of occupational groups associated with higher incomes have access to a wider array of psychosocial and other resources. Conversely, individuals who cannot rely on informal social structures when encountering problems report worse health; this effect is related to dysfunction of social structures, socioeconomic deprivation, and lack of perceived control [28], [29]. Our analysis suggests that the differences we found are not related to pre-existing medical conditions and may thus be linked to care or access to care. It is interesting to note that the converse is observed amongst infants born very preterm [30]; there are no socioeconomic differences in neonatal care or survival after very preterm birth, although mothers from the most deprived areas were nearly twice as likely to have a preterm infant than those from less deprived areas. Why mothers are less likely than their infants to receive equitable care clearly requires further investigation.

This study has highlighted two factors previously unreported as being linked with progression from morbidity to death, depressive illness and learning or intellectual disability. Women with depressive illness were over-represented by five-fold amongst the women who died from severe obstetric morbidity. Recent reports [4], [9] have highlighted suicide as an important cause of maternal death, leading to recommendations to maternity services that all women with a history of psychiatric disorder should be identified at their first antenatal visit, and that women with a previous history of serious affective disorder or other psychoses should be referred in pregnancy for psychiatric assessment and management even if they are currently well. It has also been recommended that psychiatric services should have in place a rapid referral system for women who are pregnant or postpartum. Our findings suggest that the converse should also be the case; maternity services should have a system for rapid referral and management of women with a history of depressive illness presenting with obstetric morbidity in pregnancy, because of their higher risk of death. Although our findings suggesting a risk of death associated with intellectual disability are based on a very small number of cases, we believe there is also a place for a similar rapid referral pathway for pregnant women with learning disabilities, alongside individually personalised antenatal care for this group of extremely vulnerable women.

Our analysis assumes that severe morbidity per se is a better outcome than mortality, whereas the morbidity itself may also represent a failure of management of less severe morbidity. Severe morbidity may be associated with long-term disability as a consequence of, for example, stroke associated with hypertensive disorders of pregnancy, or hypoxic brain injury following resuscitation from amniotic fluid embolism [31], [32], and it is important that every effort continues to be made to prevent both severe morbidity and mortality through, for example, strict control of blood pressure. Continued monitoring of physiological parameters in all mothers, particularly post-delivery, through the use of early warning scoring systems is now being advocated to detect and manage morbidity early in its course [9], although there is currently little evidence in pregnancy about what thresholds in physiological parameters are appropriate to trigger actions.

### Study limitations

Collection of the cases was performed nationally across the UK for both the women who died and those who survived. The UK is, to our knowledge, the only country where both the necessary data collection systems exist to allow this type of analysis. In this analysis we took advantage of data which had already been collected to allow us to rapidly investigate the progression from morbidity to death without the need to carry out a new study which would take several years to complete. However, the use of existing data has limitations. Some major causes of direct maternal death such as sepsis were not included because data were not collected by the UK Obstetric Surveillance System during the time period for which maternal death data were available. Similarly, we were unable to include cases of hemorrhage, because there were insufficient data in the CMACE database to identify cases with the same definitions as used in UKOSS. However, the specific range of conditions studied accounted for 42% of direct maternal deaths over the 2003–2008 period in the UK and cover all other major groupings of cause of death. Cases of indirect maternal death were not included; these results are therefore only generalizable to women suffering from direct obstetric morbidity. Ongoing surveillance of a full range of disorders causing both maternal mortality and morbidity would allow analysis of a wider range of conditions in the future; however, the marginal benefits of such an approach need to be weighed against the expense and burden to clinicians of reporting a much larger number of cases, compared with the current targeted approach focusing on specific morbidities in a changing programme.

UKOSS data were collected during different durations and time periods from CMACE. However all the UKOSS data were collected during the 2003–2008 CMACE data collection period and we believe that significant bias associated with these different data collection timings is unlikely. As maternal deaths are rare, this approach maximizes the statistical power of these analyses and therefore improves the validity of the results. However, the two sets of data were collected separately and therefore the number of comparable data items is limited. It is also possible that there was differential case ascertainment between the two systems, although since both systems use several methods to ensure maximal case ascertainment, we believe this is unlikely to have led to significant bias.

Data about BMI and occupational status were missing for a substantial proportion of women, particularly for those who died; we therefore performed multiple imputation analyses assuming that data were missing at random. As more data were missing in the CMACE collection we included the outcome (death or severe maternal morbidity) in the multiple imputation model as this method has been shown to provide the best results when dealing with missing data [33]. Additionally, we performed sensitivity analyses to explore the effects which may potentially bias our results; these observed associations were consistent with those found after the multiple imputation procedure. The only exception to this was the association between BMI≥30 kg/m^2^ and maternal death that was not statistically significant under all hypotheses. However, even under the most extreme hypothesis in the sensitivity analysis, the same trends were found, thus this observation may represent limited statistical power.

### Conclusions

The messages from this study can be used to inform actions to reduce maternal mortality throughout the developed world. Ongoing high quality national surveillance programmes still have an important role to play in addressing new challenges in maternal health and care. Women from vulnerable populations in high resource countries remain at increased risk of maternal death in the presence of severe maternal morbidities. This study has identified that women with a history of depressive illness and intellectual disability are over-represented amongst women who die, suggesting a need for rapid referral systems for women with these co-morbidities and pregnancy morbidity. There is a clear place for public health action to reverse the rising trends in maternal age at childbirth and clinical action to mitigate its effects, and to reduce the burden of obesity in pregnancy. Further research is needed to address weight management prior to, during and after pregnancy. In addition, development and evaluation of services to mitigate the risk of dying associated with being of black Caribbean or African ethnicity and being unemployed or from routine or manual socioeconomic groups is essential. It is not clear whether the increased risk of death is related to difficulties in access to maternal care through physical (location) or cultural factors. There is thus a place for more in depth studies to determine exactly why the presence of these factors makes women more likely to die. As the latest figures from the World Health Organisation indicate [2], even in the developed world this is no time for complacency.

## Supporting Information

Methods S1 **Definitions of Severe Acute Maternal Morbidities included in this analysis.**
(DOCX)
