# Supplementary material for: Risk Factors for Progression from Severe Maternal Morbidity to Death: A National Cohort Study
Source: PLoS One. 2011 Dec 28;6(12):e29077. doi: 10.1371/journal.pone.0029077 (PMC3247232; doi:10.1371/journal.pone.0029077)
Supplement: Methods S1 — Definitions of Severe Acute Maternal Morbidities included in this analysis. (DOCX) [file pone.0029077.s001.docx]

**Acute fatty liver of pregnancy**

- Acute fatty liver of pregnancy confirmed by biopsy.
- Or a clinician has made a diagnosis of acute fatty liver of pregnancy with signs and symptoms consistent with acute fatty liver of pregnancy present.

**Amniotic fluid embolism**

- Either a clinical diagnosis of amniotic fluid embolism (acute hypotension or cardiac arrest, acute hypoxia, or coagulopathy in the absence of any other potential explanation for the symptoms and signs observed) or diagnosis on post-mortem (presence of fetal squames in the lungs)

**Antenatal pulmonary embolism**

- Either pulmonary embolism is confirmed antenatally with suitable imaging (angiography, computed tomography, echocardiography, magnetic resonance imaging, or ventilation-perfusion scan showing a high probability of pulmonary embolism)
- Or a clinician has made a diagnosis of pulmonary embolism antenatally with signs and symptoms consistent with pulmonary embolism present, and the patient has received a course of anticoagulation treatment of more than one week’s duration

**Eclampsia**

The occurrence of convulsions during pregnancy or in the first 10 days postpartum, together with at least two of the following features within 24 hours after the convulsions:

- Hypertension (a booking diastolic pressure of <90 mm Hg, a maximum diastolic of ≥90 mm Hg, and a diastolic increment of ≥25 mm Hg)
- Proteinuria (at least protein present in a random urine sample or ≥0.3 g in a 24 hour collection)
- Thrombocytopenia (platelet count of less than 100×109/l)
- An increased plasma alanine aminotransferase concentration (≥42 IU/l)
- An increased plasma aspartate aminotransferase concentration (≥42 IU/l).

**Stroke**

All women in the UK identified as having a stroke during pregnancy were included if the stroke was:

- Confirmed by a consultant neurologist or physician
- Confirmed by diagnostic testing (e.g. MRI/CT)
